# High Resistance of a Sludge Enriched with Nitrogen-Fixing Bacteria to Ammonium Salts and Its Potential as a Biofertilizer

**DOI:** 10.3390/bioengineering8050055

**Published:** 2021-05-01

**Authors:** Claudia Rodriguez-Gonzalez, Carolina Ospina-Betancourth, Janeth Sanabria

**Affiliations:** 1 School of Environmental & Natural Resources Engineering, Engineering Faculty, Universidad del Valle, Cali 760032, Colombia; Claudia.rodriguez.gonzalez@correounivalle.edu.co; 2 School of Engineering, Newcastle University, Newcastle upon Tyne NE1 7RU, UK; m.c.ospina2@ncl.ac.uk

**Keywords:** bio-enrichment, biofertilizer, domestic wastewater treatment plants, nitrogen fixing bacteria, sequential batch reactor

## Abstract

The increasing use of chemical fertilizers causes the loss of natural biological nitrogen fixation in soils, water eutrophication and emits more than 300 Mton CO_2_ per year. It also limits the success of external bacterial inoculation in the soil. Nitrogen fixing bacteria can be inhibited by the presence of ammonia as its presence can inhibit biological nitrogen fixation. Two aerobic sludges from wastewater treatment plants (WWTP) were exposed to high ammonium salts concentrations (>450 mg L^−1^ and >2 dS m^−1^). Microbial analysis after treatment through 16S pyrosequencing showed the presence of *Fluviicola* sp. (17.70%), a genus of the Clostridiaceae family (11.17%), and *Azospirillum* sp. (10.42%), which were present at the beginning with lower abundance. Denaturing gradient gel electrophoresis (DGGE) analysis based on *nif*H genes did not show changes in the nitrogen-fixing population. Nitrogen-Fixing Bacteria (NFB) were identified and associated with other microorganisms involved in the nitrogen cycle, presumably for survival at extreme conditions. The potential use of aerobic sludges enriched with NFB is proposed as an alternative to chemical fertilizer as this bacteria could supplement nitrogen to the plant showing competitive results with chemical fertilization.

## 1. Introduction

Dinitrogen (N_2_) is one of the nutrients that limits and controls the productivity and functioning of agricultural ecosystems [1]. There are two main ways to fix nitrogen: first, through human engineered processes (54%) such as a) the Haber–Bosch process (H–B); and b) fossil fuel combustion or in a bioreactor culturing specific strains of Nitrogen-Fixing Bacteria (NFB). Nitrogen can also be fixed by natural processes (46%); for example, oxidation of nitrogen by electric storms and biological fixation [2]. Food production and industrial uses represent 79% and 21%, respectively [3]. Every year, H–B and fossil fuel digestion processes are associated with negative environmental impacts because they cause more than 1.6% CO_2_ global emissions [4,5,6]. The use of fertilizers modifies the diversity of the soil microbial community [7], water eutrophication and toxicity [8]. Additionally, soil salinization reduces natural biological nitrogen fixation and crop productivity [8,9]. Currently, different fertilization methods have been proposed as alternatives to the use of chemical fertilizers. These practices correspond to the use of soil organic amendments (which require sources of organic matter) but also of bioinoculants (plant growth-promoting rhizobacteria (PGPRs) or symbiotic fungi), in particular, isolated microorganisms [10,11]. These types of inoculants have decreased the use of chemical fertilizers by 10–20% [12,13,14] and take advantage of the microbial capacities of some soil microorganisms [15]. However, ammonium remnants and high salinity limit the persistence of bacteria and the long-term recovery of the NFB [15].

NFB have been reported in different WWTP [16,17] and isolated from municipal WWTP [18]. Municipal wastewater is characterized by high concentrations of ammonium (>50 mg L^−1^ NH_4_^+^) and salts (>2 dS m^−1^) [19]. The presence of NFB in these systems suggests they are resistant to high conditions of salinity and ammonium. These characteristics could make them suitable as alternative biofertilizers. Recently, we reported a low inhibitory effect of ammonia on the nitrogen-fixing activity of a sludge [20]. Additionally, we worked on a solution to decrease synthetic nitrogen use for biotechnological applications, for example, supplement nitrogen sources for algae cultivation, inoculum as biofertilizer, using a local microbial biodiversity implementing engineering enrichment method (data not published).

## 2. Materials and Methods

### 2.1. Inocula Collection

Three wastewater sludges (1 L) were collected from three different WWTP: (i) Activated Sludge (ASI), (ii) Biodisc (BI) and (iii) Facultative Pond (FPI). BI and FPI are located in Ginebra (3°42′40.0″ N, 76°16′16.0″ W), Valle del Cauca, Colombia. ASI is located in Cali (3°23′20.5″ N 76°30′53.9″ W), Valle del Cauca, Colombia. Sampling was done in January during the dry season. The samples were collected in a complete mix or from a biofilm depending on the corresponding system. All samples were transported at room temperature for analysis.

### 2.2. Bioreactor Operation

#### 2.2.1. Phase 1. Enrichment of NFB

Three Aerobic Sequential Batch Reactors (SBR) [21] were assembled and operated simultaneously under different inoculum origins (ASI, BI and FPI) in 1 L bottles with 600 mL working volume for 110 d at 30 °C. Each reactor was inoculated with 200 mL of wastewater sludge and fed with 400 mL of nitrogen-free medium. Sucrose (5 g L^−1^) was used as a carbon source. Each SBR operating cycle consisted of three steps: (i) Feeding with fresh nitrogen-free culture media (Ashby) [22]; (ii) Aerobic reaction time during 24 h; and (iii) Settling of biomass during 2 h followed by supernatant removal. The two inocula (ASI and FPI) that showed the highest amount of TKN were used in the next phase (Phase 2).

#### 2.2.2. Phase 2. Inocula Adaptation to Ammonium Salts Conditions

ASI and FPI were exposed to high concentrations of ammonium salts (<450 mg L^−1^). Four bioreactors distributed in two treatments that differed by the source of the inoculum were assembled and operated similarly to Phase 1 during 5 weeks. Every week, the concentration of ammonium ((NH_4_)_2_SO_4_) in the culture media was increased as follows: 0 (week 1), 50 (week 2), 150 (week 3), 300 (week 4) and 450 (week 5) mg L^−1^ NH_4_^+^. Additionally, each treatment contained one negative control reactor operated without any nitrogen source.

#### 2.2.3. Chemical Analysis

Total Kjeldahl Nitrogen (TKN), ammonium (NH_4_^+^), nitrate (NO_3_^-^) and nitrite (NO_2_^-^) were quantified from samples taken after the hydraulic retention time in complete mix (biomass + culture media) during both phases following the Standard Methods [23]. Dissolved Oxygen (DO), pH and Electrical Conductivity (EC) were quantified using a portable multiparameter *Multi 340i SET*.

### 2.3. Survey Bacterial Community

#### 2.3.1. DNA Extraction, PCR, and DGGE

0.25 gr of each inoculum was taken for nucleic acid extraction at the beginning and end of each phase. Genomic DNA extraction was performed using the Powersoil DNA Isolation Kit (MO BIO^®^, Carlsbad, CA, USA). The amplification of the *nif*H gene was made by nested PCR. The first PCR were carried out with the primer pair PoLF/PolR [24]. In the second PCR, the forward primer (PolF) contained a GC clamp, used for the denaturing gradient gel electrophoresis (DGGE). The conditions of both reactions were: preheating at 95 °C for 5 min, melting at 94 °C for 30 s, annealing at 52 °C for 30 s, elongation at 72 °C for 30 s and a final elongation at 72 °C for 10 min. PCR products (20 μL) were loaded onto 8% (*w/v*) polyacrylamide–bisacrylamide gel with denaturing gradients from 40% to 70%. Electrophoresis was run at 100 V at 60 °C for 16 h in 1 × TAE buffer. Gel revelation was performed by silver nitrate staining. The DGGE profiles were analyzed using the GelQuest and ClusterVis software [25,26].

#### 2.3.2. RNA 16S Gene Sequencing and Diversity Analysis

Bacterial DNA was amplified with tagged primers 27F/519R covering V1–V3 region of 16S gene from the samples at the 0 and 35 days of Phase 2 [27]. A single-step 30 cycle PCR were used under the following conditions: 94 °C for 3 min, followed by 28 cycles of 94 °C for 30 s; 53 °C for 40 s and 72 °C for 1 min; after which a final elongation step at 72 °C for 5 min was performed. All amplicon products obtained from different samples were mixed in equal concentrations and purified using Agencourt Ampure beads (Agencourt Bioscience Corporation, Beverly, MA, USA). Sequencing of the DNA was performed at Mr. DNA laboratory (Shallowater, TX, USA) using a Roche 454 FLX titanium instrument, following the manufacturer’s guidelines.

Raw data were independently processed and quality-filtered (q > 25, reads > 150 bp) using QIIME software 1.9.0 [28]. Operational Taxonomic Units (OTUs) were defined by clustering at 3% divergence (97% similarity). Finally, OTUs were taxonomically classified using the uclust method against a curated database derived from SILVA (release 132) [29]. To compare community structures, a maximum likelihood phylogenetic tree and rarefaction curves using α diversity were constructed. All the found bacteria were compared with UniProt UniProtKB/Swiss-Prot database [30] to identify NFB.

### 2.4. Evaluation of Biofertilization

The sludge adapted (ASI) to high ammonium salts was as a biofertilizer for coriander plants (*Coriandrum sativum*) in a greenhouse (day temperature of 24 °C, relative humidity of 73%). The sludge adapted was compared with a common biofertilizer based on *Azotobacter chroococcum* and chemical fertilizer based on brand. Commercial *Azotobacter chroococcum* is available in liquid culture media with a population concentration of 1 × 10^8^ UFC mL^−1^. The doses of adapted sludge and commercial biofertilizer were 0.092 mL and 0.106 mL per plant, respectively. For chemical fertilization, 1.34 g CH_4_N_2_O, 1.00 g (NH_4_)_3_PO_4_ and 0.63 g KCl were used. All the amounts used for the fertilization types were dissolved into 91 mL d^−1^ of distilled and sterile water per plant of the irrigation sheet. A degraded soil from sugar cane crops (8.54 dS m^−1^) in Valle del Cauca, Colombia, was used. Four coriander seeds (*Coriandrum sativum*) were planted in pots (30 cm of diameter) with 2 kg of soil and 30 min later they were irrigated with the fertilizer type tested in the study, adapted sludge (ASI), commercial biofertilizer or chemical fertilizer, respectively.

Six treatments were replicated five times as follows: (T0) Plants were left free from any fertilization and served as a control. (T1) Plants were treated with the commercial inoculum (*Azotobacter Chroococcum*, 15 L Ha^−1^). (T2) Plants were treated with commercial chemical fertilizer (1.34 g CH_4_N_2_O, 1.00 g (NH_4_)_3_PO_4_ and 0.635 g KCl). (T3) Plants were treated with the adapted inoculum (−10% of total dose, 0.083 mL). (T4) Plants were treated with the adapted inoculum (total dose, 0.092 mL). (T5) Plants were treated with the adapted inoculum (+10% of total dose, 0.101 mL). The plants in each group were allowed to grow up for 90 days and then harvested to determine the plant growth parameters including dry weight, plant height, number of flowers per plant and weight of yield per plant, as well as foliar total nitrogen (%), were measured.

## 3. Results

### 3.1. Phase 1. Enrichment of Nitrogen Fixing Bacteria

**Figure 1:**
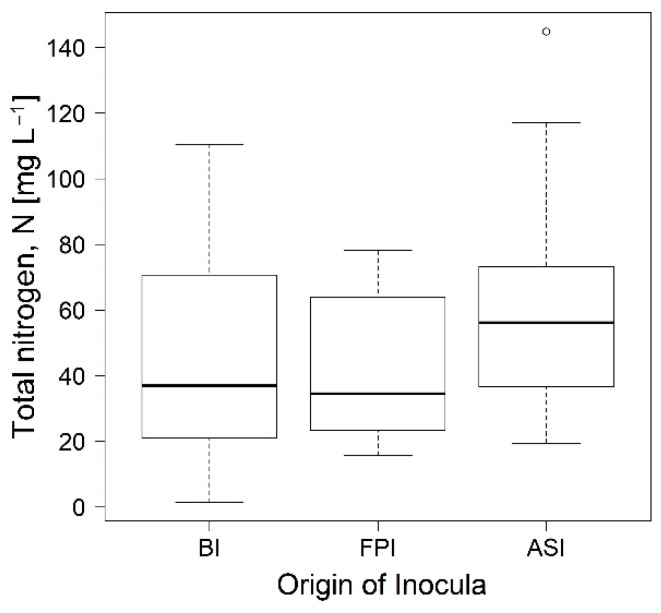
Total nitrogen found in the bioreactors inoculated from Activated Sludge (ASI), Biodisc (BI) and Facultative Pond (FPI). “°” Boxplot outliers.

Considering the average values of pH and DO (7.11 units, 2.92 mg L^−1^ DO) during the bioreactors operation, losses as NH_3_ would not occur [31]. Considering that culture media are nitrogen free, an increase of TKN due to biological nitrogen fixation was observed in all reactors in the experiments. Figure 1 shows the amount of total nitrogen in the three bioreactors. No significant differences were found between reactors (*p*-value = 0.69) inoculated with different sources of sludge. The bioreactor that contained the lowest amount of nitrogen was FPI (42.18 mg L^−1^ TKN), while the bioreactor that contained the highest amount of nitrogen was ASI (59.51 mg L^−1^ TKN).

**Figure 2:**
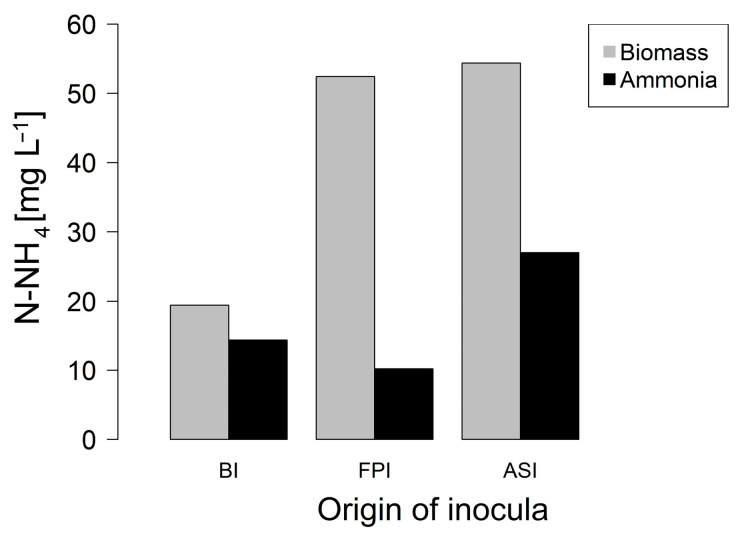
Biomass (gray) and effluent (black) N-NH_4_^+^ content of (BI): Biodisc, (FPI): Facultative Pond and, (ASI): Activated Sludge.

Table 1 shows the increase of nitrogen compounds measured. Purges and washing were done to avoid nitrogen accumulation in the bioreactor due to the fast biomass increase. The biomass concentration remained after the purge process was 25–38 mg L^−1^ of TKN. Figure 2 shows the biomass and supernatant N-NH_4_^+^ content at the end of Phase 1. The biomass:supernatant NH_4_^+^ ratio at the end of the experiment was: BI = 1.35; FPI = 5.14; ASI = 2.01. The reactors with a major biomass and ammonia content were ASI and FPI which were carried on to the next experiment (Phase 2).

### 3.2. Phase 2. Inocula Adaptation to Ammonium Salts Increase

**Figure 3:**
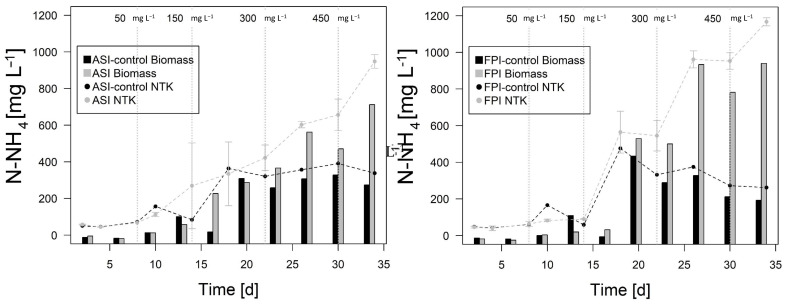
Total N-NH_4_ (dot plot) and Biomass (bar plot) content found in the bioreactors (**a**) ASI and (**b**) FPI. ASI-control: Activated Sludge Control. FPI-control: Facultative Pond Control.

FPI and ASI were exposed to a progressive increase of ammonia salts in the culture media with concentration from 0 up to 450 mg L^−1^ (4.45 dS m^−1^ equivalent) (in local degraded soil the range of residual ammonia varies between 0–50 mg L^−1^ and 2–5 dS m^−1^ of salinity). Figure 3a,b shows the biomass concentration related to the increase of N-NH_4_ concentration in the bioreactors. A constant increase of biomass was observed in ASI, while during the first 21 days, FPI did not show significant changes. After day 21, the increase of ammonium concentration did not have any effect on the response of the biomass and no significant differences between the bioreactors and their controls were observed. The ammonium in the supernatant started to increase sharply after 21 days (300 mg L^−1^ NH_4_^+^).

### 3.3. Microbial Diversity Analysis with DGGE

**Figure 4:**
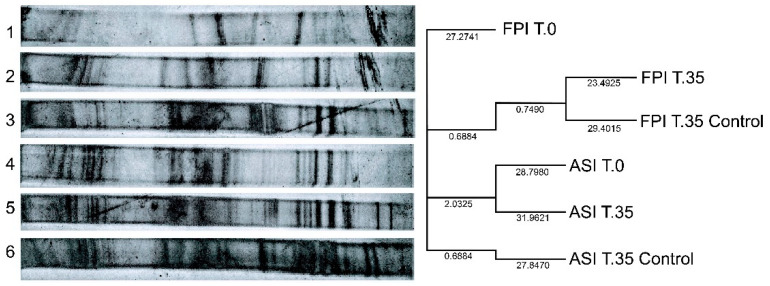
Pearson–Phi correlation coefficient distance cladograms generated from the DGGE fingerprints profile of *nif*H gen of (**1**) FPI Phase 2-Initial time (T.0); (**2**) FPI Phase 2-end (T.35); (**3**) FPI Control-end (T.35); (**4**) ASI Phase 2-Initial time (T.0); (**5**) ASI Phase 2-end (T.35); (**6**) ASI Control-end (T.35). Detected by silver-nitrate staining.

NFB diversity in the sludge ASI and FPI were analyzed using DGGE profiles. This analysis was performed at the beginning (T.0) and end (T.35) of Phase 2. Cluster analysis was performed using the Pearson Phi correlation coefficient with Sequentix ClusterVis software [26]. Phylogenetic analysis with Neighbor Joining trees was used. Figure 4 shows the results of DGGE diversity analysis. All the samples (ASI, FPI and their controls) were *nif*H gene positive. There are not significant differences between the exposed salts inocula and their controls.

No significant differences were found between NFB of ASI T.0 and ASI T.35. However, there could been an increase or decrease of the relative abundance of some species. A similar situation occurs with FPI T.0 and FPI T.35. There was more diversity of NFB in ASI.

### 3.4. 16S rDNA Diversity Analysis

**Figure 5:**
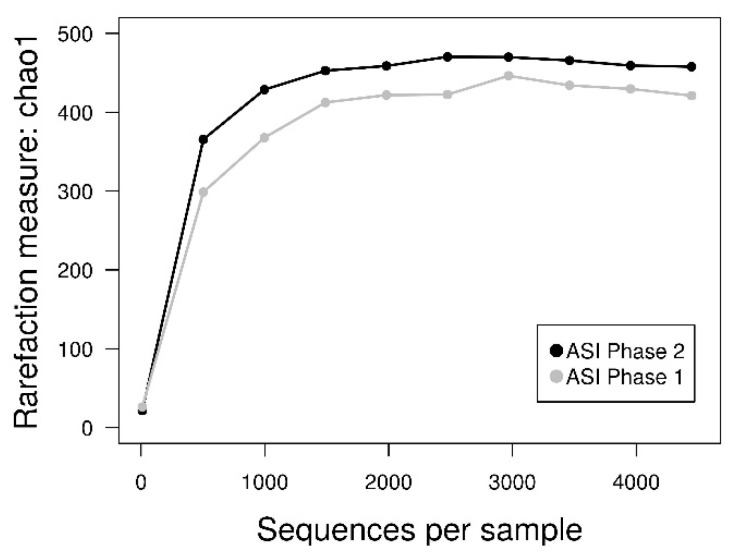
Rarefaction plots of ASI on Phase 1 (gray) and Phase 2 (black).

The inoculum that showed most microbial diversity with DGGE profiles (ASI in Phase 1 and Phase 2), the 16S rRNA gene was sequenced. At the end of Phase 1 and Phase 2, samples from the ASI reactor were sequenced. A total of 16,536 assembled sequence reads were obtained. These sequence reads were assigned to a total of 607 OTUs. Observed OTUs belonged to 12 phyla among all samples; dominant phyla included Bacteroidetes (44%), Proteobacteria (30.89%), Firmicutes (13.59%) and Actinobacteria (4.79%). Rarefaction curves were generated using QIIME 1.9.0. At 3% of the genetic distance, rarefaction curves show that the sampling effort was substantial. Bacterial richness was completed reaching the curvilinear or plateau phases at approximately >1500 sequences per sample (Figure 5). ASI at Phase 2 had more diversity than Phase 1 with no significant differences.

**Figure 6:**
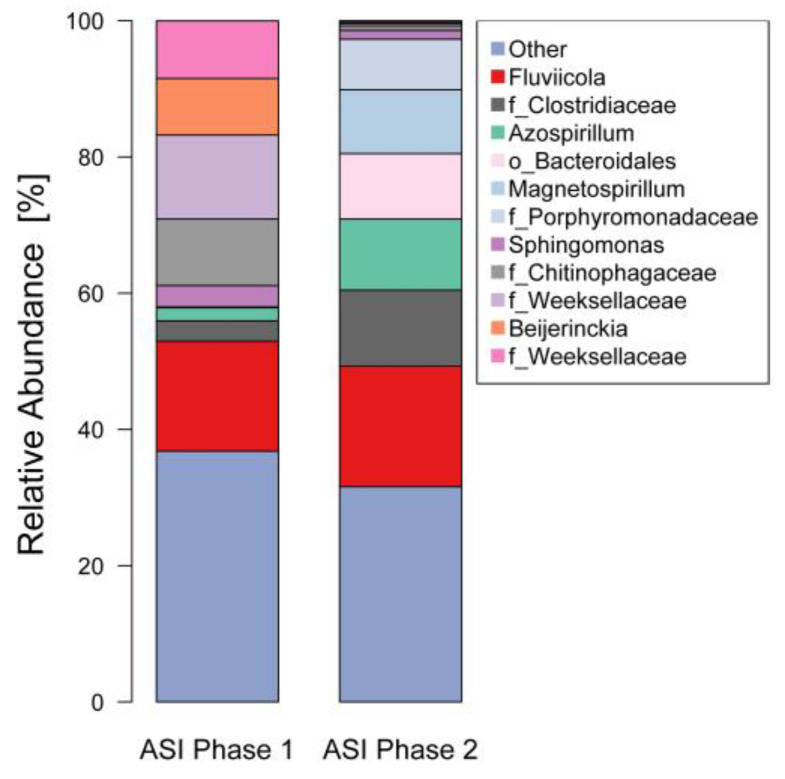
Genus relative abundance in the bioreactor ASI at the end of Phase 1 and 2 (most representative < 1%).

The most representative genera (>1%) in the inocula were *Fluviicola* sp. (17.70%), a genus of the Clostridiaceae family (11.17%), *Azospirillum* sp. (10.42%), a genus of Bacteroidales orden (9.60%), and *Magnetospirillum* sp. (9.37%) (Figure 6). The beta-significance analysis compares the existence of statistical differences between two samples. When the microbial community beta-significance of the inoculum ASI in Phase 1 and Phase 2 was compared, there were no significant differences (*p* value = 0.081). The beta-diversity analysis, using the dissimilitude parameter weighted unifrac [32], shows that the inoculum is about 20% dissimilar. Therefore, it can be concluded that the exposure to high (450 mg L^−1^) ammonium salts, did not create significant changes to the community.

### 3.5. Biofertilizer Effect on Coriandrum Sativum

The field trials of microbial fertilization on coriander plants showed significant increase in organic matter content, total nitrogen content and ammonium in soil when compared with the control. On the other hand, there are not significant differences in plant properties; however, foliar nitrogen content with microbial fertilization is comparable to chemical fertilization (Table 2 and Table 3).

## 4. Discussion

The metabolic functions of nitrogen transformation were conserved despite differences in biodiversity (origin of inocula). Those slight differences in the bioreactors suggest a differed ecological adaptation of the consortia at the given conditions at Phase 1. The amount of total nitrogen found in each bioreactor was higher compared to the results obtained by Pratt et al. [33], which achieved a production of 27 mg L^−1^ of TKN using inocula from paper treatment sludge and acetate as an unique carbon source. Additionally, more recently, Welz et al. [34] enriched nitrogen fixing bacteria in a sand bioreactor fed with winery wastewater, reaching a maximum of 12 mg L^−1^ total nitrogen. The nitrogen available for the metabolism was produced by NFB and employed in a different pathway. NFB allowed the presence of other microbial groups involved in the nitrogen cycle such as nitrifying and denitrifying bacteria. These possible metabolic associations can lead to consortia formation making matter and energy transfer more efficient. Despite not being able to find evidence of the presence of nitrifying microorganisms by dependent or independent culture technique, the bioreactors were under conditions that favored nitrification such as >2 mg L^−1^ DO and 6.5–7.5 pH [34]. The consumption of nitrite and production of nitrate and ammonia suggest the presence of this nitrifying bacteria. Purges and washing were done to avoid nitrogen accumulation in the reactor media and saturation due to the fast biomass increase.

At the Phase 2, the inocula FPI and ASI were exposed to an ammonia salts concentration increase from 0 to achieve 450 mg L^−1^ (4.45 dS m^−1^ equivalent). The ammonium was not completely consumed, due probably to the imbalance of the C/N ratio [35]. According to Pratt et al. [33], the C/N ratio must be approximately 100:3.5 in order to assure the uptake of the nitrogen source. This finding additionally suggests the presence of mixotrophic bacteria; when they have nitrogen available in the culture media, they will use it and the nitrogenase activity will be inhibited, but when there is no nitrogen available in the culture media, they will fix it from the atmosphere. The salinity can be measured as Electrical Conductivity (EC). Normally, a degraded soil presents an EC greater than 2 dS m^−1^. Specifically, for the case of sugarcane crops, an EC more than 2 dS m^−1^ represents a reduced overall productivity of 7% [36]. The EC on the bioreactors at the beginning of the SBR cycle was around 1.33 dS m^−1^ and at the end was 2.23 ± 0.92 dS m^−1^. Hence, the enriched inocula could adapt successfully when exposed to conditions similar to the ones of degraded soil.

Diazotrophic bacteria diversity on the inocula ASI and FPI were analyzed by PCR-denaturing gradient gel electrophoresis (DGGE). The presence of NFB across the treatments showed that these bacteria were conserved. Nevertheless, the thickness of the band was greater after enrichment. The environmental and operational conditions in which the inocula were exposed could have affected the diazotrophic population, but it still conserved the majority of nitrogen fixing populations, as 92.6% of the fingerprints in ASI were conserved at the end of the experiment (compared to samples taken at the beginning). The presence of NFB in all the inocula demonstrated that the increasing concentration of ammonium salts was not an inhibitory factor for the NFB population. The successful adaptability of this sludge to stress condition makes it a sustainable alternative for biotechnological applications, such as a biofertilizer in degraded soils with excess ammonium salts (450 mg L^−1^).

### 4.1. Microbial Associations in the Bioreactor

Possible microbial associations between the most representative genera were identified with the aim of getting an approximation of their role in the community. These genera were compared against the UniProt database (updated 2018) using the enzymes involved in the nitrogen cycle [1,37]. The nitrogenase enzyme was present in the inocula and 54.42% of the genera were reported as NFB. The three most representative genera of NFB were *Fluviicola* (17.70%), *Azospirillum* (10.42%) and *Magnetospirillum* (9.37%). It is likely that these microorganisms incorporated the nitrogen into the community. Regarding the genera associated with the nitrogen cycle enzymes, it suggests the association of different clusters of microorganisms, where each group has various functions involved in the nitrogen cycle. The most representative consortia or cluster probably is formed between the genus *Fluviicola*, family Clostridiaceae, and *Magnetospirillum*. *Fluviicola* is present in freshwater [38] and wastewater [39]. This genus is strictly aerobic, Gram-staining-negative, non-flagellated rods with motile by gliding and utilizes carbohydrates for growth [38]. Thus, this genus is probably located in the external part of the cluster, taking up the primary carbon source (saccharose), and is exposed to aerobic medium conditions.

*Magnetospirillum* has been described as oxidase and catalase positive. Oxidases enzymes are important in the nitrification process [1,37]. *Magnetospirillum* could be involved in the nitrifying process and it uses organic acids as a carbon source; however, it is not common for it to use carbohydrates. *Magnetospirillum* could be within the cluster using the ammonia already fixed from the external part and taking up another carbon source as the energy source. On the other hand, the genus *Azospirillum* is one of the most important nonsymbiotic and microaerobic NFBs. This aerotactic behavior is necessary to guide the bacteria to optimal niches for microaerobic nitrogen fixation. The sensory system for spatial oxygen and redox gradients characterized in *Azospirillum* is unique. *Azospirillum* fixes nitrogen only under microaerobic conditions. Additionally, the adaptation of genus *Azospirillum* to stress salinity conditions has been reported by Fukami [40]. The presence of *Azospirillum* genus as one of the most representative bacteria in the adapted inocula suggests the association with *Fluviicola,* for example, in the microaerobic environment of the cluster.

The Clostridiaceae family is strictly anaerobic and chemotrophs. Its principal genus is *Clostridium*. This bacteria is possibly located in the internal part of the association and together with other microorganisms as *Fluvicola*, *Azospirillum* and *Magnetospirillum*. The genus *Paludibacter* has been reported as strictly anaerobic, and in this environment, the association with other microorganisms is the key for the adaptation and could be associated with the denitrification process. It is possible that the remaining 42.86% of bacterial species were not associated with the nitrogen cycle but we propose that they are related to the carbon cycle, for example, saccharose transformation via aerobic and anaerobic pathway. Less abundant genera such as *Paenibacillus* (1.47%), *Pseudoxanthomonas* (1.37%), *Rhodobacter* (1.12%) or *Xanthobacter* (1.00%) are reported as nitrogen-fixing bacteria and could be within the cluster together with other less representative bacteria. In summary, the inocula contains different associations between the microorganisms that allow the adaptation to stress salinity conditions. No significant changes in the community were found.

### 4.2. Inoculum Application in Soil

In order to apply the adapted inocula in degraded soil conditions, an experiment was carried out using coriander plants. Coriander plants have a of lot medical and gastronomy uses. It is s a common spice and a major curry powder ingredient and contains antimicrobial, hypolipidemic, hypoglycemic action and insecticidal properties [41] due to the presence of phenolic compounds (lignins and flavonoids) mainly located in the leaves [41]. Therefore, number of leafs and foliar nitrogen are the most important properties. The results show a positive response of the different treatments using the adapted inocula. Inhibition by heavy metals accumulation was not appreciable. The concentrations of Organic Matter Content (OMC), Total Nitrogen Content (TNC) and NH_3_ in T5 were increased, 1.53%, 0.14% and 38.6 mg kg^−1^, respectively. The OMC, NH_3_ and TNC in the treatments T1–T4 did not have significant differences. It is evident that the bio-fertilization process allows nutrient liberation and improvement of soil properties. All the treatments with microbial fertilization (T4–T5) showed an increase of OMC and TNC compared with the control (T0 and T1) and similar results compared with chemical fertilization (T2). The ammonia found in the soil suggests that there was biological nitrogen fixation together with other microbial transformation processes involved in the nitrogen cycle. The increase of ammonia found in the soil (Table 2) suggests nitrogen fixation activity and not ammonia released from organic matter decomposition.

Studies have shown that biofertilizer such as PGPRs together with salinity stress affect the growth, yield and physiology of coriander plants [14]. In our experiments, lower doses of adapted inoculum (T3) showed better plant properties and similar results to chemical fertilization (T2). The results suggest that less concentrations of inoculum allow a better interaction between plant and inoculum avoiding nutrient competition by trophic groups. Treatments with the adapted inoculum (T3–T5) showed lower flower production when compared to the control (T0). This trait is desirable for human coriander consumption but it can also be associated with soil plant stress due to salinity conditions. On the other hand, the height of plants is not directly correlated with type of fertilization [14].

In this paper, we proposed an alternative way of sludge as a source of adapted biofertilizer. We need innovative technologies and waste recovery plays an important role [42]. Sewage sludge can be used in agriculture after stabilization and removal of toxic compounds and pathogens and undesirable odors [42]. However, human risk by pathogen must be studied in depth. The results found in the coriander plants suggest that there was not a high risk of use of these inocula, as long as there is a microbiological selection process. Some studies suggest that the use of sludge did not show a negative impact on the yield quality or soil quality [43,44]. However, it is necessary to contemplate the evaluation of the stability of this inoculum in the soil. The biggest threat is the presence of micro-pollutants, including heavy metals and pathogenic bacteria, which must be removed before real application [45]. Selection and bioprospection by bioreactors seem to be the proper way of sludge management.

It is important to point out that bacterial species that were enriched in the reactors such as *Magnetospirillum*, *Fluviicola* and *Azospirillum* are non-pathogenic NFB which could be ideal as biofertilizer. These bacteria were able to survive under a high ammonia condition; however, we do not yet know the underlying mechanisms that allowed their endurance. Future studies should investigate if the tolerance to ammonia within the reactor is given by individual physiological conditions of the bacterial species (in order to promote their cultivation and application as pure culture) or due to consortia associations. We believe that it is possible to solve these inquiries by reconstruction of specific nonpathogenic consortia using the information of the bacterial species with this previous enrichment.

In this paper, we sought to select microorganisms that are resistant both to ammonium and nitrogen fixers; thus, that have the potential to survive in agricultural soils containing nitrogen-based chemical fertilizers. This type of bacteria could be found in substrates “contaminated” with sewage wastewater or residues from the pork poultry industry. Although we could not see an enrichment of pathogenic NFB in our sludge, we cannot guarantee that they were not present in lower abundances. Our proposal for a new strategy of nitrogen fixation is a valid approach for the *bioprospection* and selection of non-pathogenic microorganisms that have developed a resistance to survive under ammonium conditions with time. This selected sludge containing bacterial consortia can function as a source of biofertilizer with a low public health risk.

## 5. Conclusions

High ammonium salts (>50 mg L^−1^) concentrations did not affect in a negative way the presence of Nitrogen Fixing Bacteria population. The resistance of this type of sludge to stress conditions make them an adaptable inoculum for application as a biofertilizer in soils containing ammonium. The methodology employed in the enrichment and adaptation of these conditions could be a way to obtain an alternative sustainable bio-product. The adapted inocula improved the soil and plant properties on *Coriander sativum*, showing it to be a promising source of inocula for further biotechnological applications. This work is the start of a research line in the valorization of native biodiversity found in the sludge of wastewater treatment plants. The results obtained in this study hint at the benefits of further exploration of the microbial biodiversity bioprospecting and inocula and engineering from ammonia-rich sludges. In the context of global environmental changes, new ways to do prospection and technological application of mixed culture are needed. It is important, in addition, to advance in the research of safe-consortia design. The utility of bioreactors to achieve this goal, especially in undeveloped countries, where genetic engineering procedures and applications are limited, has been demonstrated.

## Figures and Tables

**Table 1:** Initial and final chemical characterization in bioreactors: DO, NH_4_^+^, NO_3_^−^, NO_2_^−^ (mg L^−1^) and pH (units).

Parameter	Inocula					
	BI		FPI		ASI	
	To	Tf	To	Tf	To	Tf
DO_R_	2.93–3.44		2.92–2.93		2.99–3.04	
pH_R_	6.70–7.33		6.84–7.70		5.67–7.67	
NH_4_^+^	4.00	13.05	3.50	11.25	2.70	13.2
NO_2_^−^	0.22	1.07	0.19	0.51	0.23	0.62
NO_3_^−^	3.24	9.73	3.91	11.74	3.03	9.10

Biodisc (BI), Facultative Pond (FPI) and Activated Sludge (ASI) inocula. Dissolved Oxygen range (DO_R_). pH_R_ range. Initial concentration of Phase 1 (To). Final concentration of the phase 1 (Tf).

**Table 2:** Effects of biofertilization on *Coriander sativum*. Soil properties.

Parameters	T0	T1	T2	T3	T4	T5
pH (units)	7.9	8.0	7.8	7.8	7.8	7.9
OMC (%)	4.03	4.41	4.32	4.70	4.65	4.41
TNC (%)	0.31	0.38	0.34	0.35	0.42	0.45
C/N	13.00	11.60	12.71	13.43	11.08	9.80
NH_3_ (mg kg^−1^)	35.0	35.9	35.9	39.7	76.1	52.7

OMC: Organic Matter Content, TNC: Total Nitrogen Content.

**Table 3:** Effects of biofertilization on *Coriander sativum*. Plant properties.

Parameters	T0	T1	T2	T3	T4	T5
No. of plants	16	16	21	19	17	19
Plant height (cm)	23.85	21.65	23.69	21.28	21.48	23.18
No. of flowers	52	27	62	42	34	44
No. of leafs	73	73	136	123	59	88
Dry matter (g)	1.04	1.12	1.96	1.56	0.74	1.22
Foliar nitrogen (%)	1.03	1.62	2.05	1.92	1.74	2.08

## Data Availability

No new data were created or analyzed in this study. Data sharing is not applicable to this article.

## Abbreviations

ASI	Activate Sludge Inoculum
BI	Biodisc Inoculum
DGGE	Denaturing Gradient Gel Electrophoresis
DO	Dissolved Oxygen
EC	Electrical Conductivity
FPI	Facultative Pound Inoculum
H-B	Haber Bosch process
NFB	Nitrogen Fixing Bacteria
OMC	Organic Matter Content
OTUs	Operational Taxonomic Units
PCR	Polymerase Chain Reaction
PGPRs	Plant Growth Promoting Rhizobacteria
SBR	Sequential Batch Reactor
T0	Treatment with no fertilization
T1	Treatment with commercial *Azotobacter chroococcum*
T2	Treatment with chemical fertilizer
T3	Treatment with adapted inocula, −10% of dose
T4	Treatment with adapted inocula, 100% of dose
T5	Treatment with adapted inocula, +10% of dose
TAE	Tris base, Acetic acid and EDTA solution
TKN	Total Kjeldahl Nitrogen
TNC	Total Nitrogen Content
WWTP	Waste Water Treatment Plant
